# Self-reported changes in drug use behaviors and syringe disposal methods following the opening of a supervised injecting facility in Copenhagen, Denmark

**DOI:** 10.1186/1477-7517-11-29

**Published:** 2014-10-28

**Authors:** Elizabeth N Kinnard, Chanelle J Howe, Thomas Kerr, Vibeke Skjødt Hass, Brandon DL Marshall

**Affiliations:** Department of Behavioral and Social Sciences, Brown University School of Public Health, 121 South Main Street, Box G-S-121-4 Providence, RI 02912 USA; Department of Epidemiology, Brown University School of Public Health, 121 South Main Street, Box G-S-121-2, Providence, RI 02912 USA; Faculty of Medicine, University of British Columbia, 317 - 2194 Health Sciences Mall, Vancouver, BC V6T 1Z3 Canada; Urban Health Research Initiative, British Columbia Centre for Excellence in HIV/AIDS, 608 – 1081 Burrard Street, Vancouver, BC V6Z 1Y6 Canada; The Saxo Institute, Faculty of Humanities, University of Copenhagen, Karen Blixens Vej 4, DK-2300 Copenhagen, Denmark

**Keywords:** Supervised injecting facility, Drug consumption room, People who inject drugs, Injection drug users, Harm reduction, Risk behaviors, Syringe disposal, Denmark

## Abstract

**Background:**

In Denmark, the first standalone supervised injecting facility (SIF) opened in Copenhagen’s Vesterbro neighborhood on October 1, 2012. The purpose of this study was to assess whether use of services provided by the recently opened SIF was associated with changes in injecting behavior and syringe disposal practices among people who inject drugs (PWID). We hypothesized that risk behaviors (e.g., syringe sharing), and unsafe syringe disposal (e.g., dropping used equipment on the ground) had decreased among PWID utilizing the SIF.

**Methods:**

Between February and August of 2013, we conducted interviews using a survey (in English and Danish) with forty-one people who reported injecting drugs at the SIF. We used descriptive statistics and McNemar’s test to examine sociodemographic characteristics of the sample, current drugs used, sites of syringe disposal before and after opening of the SIF, and perceived behavior change since using the SIF.

**Results:**

Of the interviewed participants, 90.2% were male and the majority were younger than 40 years old (60.9%). Three-quarters (75.6%) of participants reported reductions in injection risk behaviors since the opening of the SIF, such as injecting in a less rushed manner (63.4%), fewer outdoor injections (56.1%), no longer syringe sharing (53.7%), and cleaning injecting site(s) more often (43.9%). Approximately two-thirds (65.9%) of participants did not feel that their frequency of injecting had changed; five participants (12.2%) reported a decrease in injecting frequency, and only two participants (4.9%) reported an increase in injecting frequency. Twenty-four (58.5%) individuals reported changing their syringe disposal practices since the opening of the SIF; of those, twenty-three (95.8%) reported changing from not always disposing safely to always disposing safely (McNemar’s test *p*-value < 0.001).

**Conclusions:**

Our findings suggest that use of the Copenhagen SIF is associated with adoption of safer behaviors that reduce harm and promote health among PWID, as well as practices that benefit the Vesterbro neighborhood (i.e., safer syringe disposal). As a public health intervention, Copenhagen’s SIF has successfully reached PWID engaging in risk behavior. To fully characterize the impacts of this and other Danish SIFs, further research should replicate this study with a larger sample size and prospective follow-up.

## Background

Supervised, or safer, injecting facilities (SIFs) — also known as supervised injecting rooms (SIRs), supervised injecting sites (SISs), and drug consumption rooms (DCRs) — have historically been implemented in response to public health and safety concerns that arise from street-based injecting in urban areas [1]. The primary objectives of SIFs are to reduce morbidity and mortality that would otherwise occur from syringe sharing, public injecting, and other activities [1], specifically infectious disease transmission and fatal overdose, and to connect people who inject drugs (PWID) to various health and social programs. These facilities are often viewed as complementing other harm reduction interventions [2], by allowing for the consumption of pre-obtained illicit substances in the most hygienic way possible under the supervision of healthcare professionals (e.g., nurses), and thus resulting in improved health and social equity for PWID [3, 4].

SIFs around the world typically provide a calm, well-lit, health-oriented venue to inject, as well as access to clean injecting equipment, referrals to external services, and emergency assistance in the event of an overdose. Some SIFs are highly comprehensive in their approach [2, 5], providing a host of medical, legal, and social services, such as extensive counseling, subsidized food, showers, lockers, and laundry services [1, 2, 4–6]. Overall, by addressing addiction as a chronic, relapsing health condition, SIFs have been found to reduce barriers to accessing health-centered care for PWID [7–11].

Beyond promoting the health of PWID, SIFs have also proven beneficial for the larger communities surrounding them. Municipalities that have implemented SIFs have seen reduced rates of drug injection in public spaces, reduced burden of illegal drug use on the community, and expanded opportunities to work with PWID [2, 12, 13]. Specifically, results from Sydney, Australia and Vancouver, Canada have shown reduced public injecting and discarded needles, as well as decreased drug overdose mortality rates in neighborhoods in which SIFs are located [11, 13–18]. Moreover, the European Monitoring Centre for Drugs and Drug Addiction (EMCDDA) has published two comprehensive reports on SIFs, concluding that they reach and are accepted by vulnerable target populations, reduce high-risk drug using behavior, prevent drug use in open spaces and related public disorder, and improve the health of PWID, and are thus recommended as part of local public health strategies [3, 14, 19].

Gaining broad-based community support for SIFs is frequently a key component of implementing these interventions [2]. This was true in Denmark, where there existed a clear need to reduce mortality rates among PWID, and to reduce public injecting and discarded paraphernalia [20–22]. In September 2011, *Fixerum*, a nongovernmental organization (NGO), opened a mobile SIF in Copenhagen, which provided services for PWID without police or government interference for ten months [14]. In June 2012, the Danish parliament adopted a new amendment (Executive order no. 606) to an existing law on psychoactive substances that gave municipalities a legal mandate allowing them to implement and operate standalone SIFs, with permission from the Minister of Health [14, 23]. The Danish parliament included language in the new law that instructed police and prosecutors not to search, seize, and prosecute users who were in possession of “small quantities” of controlled substances for personal use “in and nearby” SIFs [24]. Copenhagen’s first standalone (non-mobile) SIF was subsequently established on October 1, 2012 in the Vesterbro neighborhood, where drug dealing and public injection drug use have historically been concentrated [20, 25]. Users of the SIF are restricted to injecting inside the facility, but clients are permitted to smoke or sniff illicit drugs in the designated outside area; thus, Danes often refer to the greater health facility as a more all-encompassing drug consumption room (DCR) [26].

To date, no peer-reviewed article evaluating behaviors and practices among PWID accessing the Copenhagen SIF, and how the facility may affect the greater Vesterbro community, has been published. However, Mændenes Hjem (the Men’s Home) in Copenhagen has conducted a survey administered among users of the SIF to investigate their experiences and satisfaction with the facility [27]. The purpose of our study was to assess whether use of the services provided by the recently opened SIF in Copenhagen was associated with changes in injecting behavior and syringe disposal practices among PWID. We hypothesized that risk behaviors (e.g., syringe sharing), and unsafe syringe disposal (e.g., dropping used equipment on the ground) decreased among PWID utilizing the SIF.

## Methods

### Study population

Members of our research team approached individuals seated in the outdoor area adjacent to the SIF to ask if they would be willing to take part in a study regarding their use of SIF services. Due to a limited timeframe for primary data collection in Copenhagen, convenience sampling approaches were employed. Eligible persons were those who reported having injected drugs at the SIF in Copenhagen, Denmark at least once since its opening on October 1, 2012. Participants were excluded if they only used drugs at the Copenhagen SIF via routes of administration other than injecting (i.e. snorting, smoking, etc.). Individuals who were eligible and agreed to participate in the study were asked to complete a survey aimed at gaining insight into injecting behaviors and syringe disposal practices prior to and following the opening of the SIF. Individuals who completed the survey were asked to refer their acquaintances who also used the SIF for study participation. All interviews were conducted between February and August of 2013.

### Survey measures and administration

Participants completed a structural behavioral survey in Danish or English, and either self-administered the survey or were interviewed by a member of the research team. If participants chose to self-administer the survey, a research assistant was available to answer any questions. All interviews were completed in person at the SIF, directly outside the building in an adjacent sitting area. Privacy during the interview sessions was ensured to the greatest extent possible by sitting in a secluded section of the outdoor seating area. At any point, participants could choose to skip a question or terminate the interview. Participants were given consent forms explaining the goals of the study, with contact information for the project supervisor at Brown University.

The survey included questions about sociodemographic characteristics, current drugs used, most common sites of injecting, frequency of use at the SIF, quality of relationships with the community and the police, as well as other activities and behaviors (e.g., overdose). We assessed the presence and absence of injecting risk behaviors such as rushed/stressful injecting, outdoor injecting, needle and syringe sharing, cleaning injection site(s), difficulty in finding a vein, reusing one’s own needles/syringes, using clean water to inject, and requiring assistance injecting. Specifically, we asked participants whether they felt they had changed any of their reported injecting behaviors that they practiced before using the SIF. Finally, participants were also asked whether they felt their frequency of injecting had changed since using the SIF.

We also assessed primary sites of syringe disposal before and after the opening of the SIF to determine whether use of the SIF was associated with safer syringe disposal methods. Specifically, if participants endorsed one or more of the following choices when asked about primary sites of syringe disposal — returned them (syringes) to the needle exchange (or SIF), put them in my own sharps container, or put them in an outdoor sharps container — they were coded as “always safe”. If they endorsed any of the following choices — threw them in the garbage, dropped them on the ground, gave them to another user, flushed them down the toilet, or other —they were coded as “not always safe.” This method of coding was used to produce two dichotomous variables — which indicated “always safe” vs. “not always safe” *before* the opening of the SIF, and “always safe” vs. “not always safe” *after* the opening of the SIF.

### Statistical analysis

Descriptive statistics were used to examine demographic characteristics of the sample, current drugs used, enrollment in treatment in the last six months, frequency of use at the SIF, and primary site(s) of injecting before the SIF opened. Next, sites of syringe disposal before and after the opening of the SIF were compared to assess whether participants’ primary sites of syringe disposal changed following the facility’s opening. McNemar’s test was used to assess whether participants who reported changing their syringe disposal practices were significantly more likely to change from “not always safe” to “always safe” after the SIF’s opening. Finally, we evaluated perceived behavior change and perceived frequency change using descriptive statistics. All *p*-values are two-sided at α = 0.05, and all analyses were conducted in SPSS (version 22.0).

This research was conducted as part of a Global Independent Study Project (GLISP) through Brown University. Based on Brown University’s Human Research Protection Program Policy and Procedure Manual, Section 11, (http://www.brown.edu/research/human-research-protection-program-policy-and-procedure-manual-section-11), this research project did not meet the definition of research (i.e. the data collected was part of an undergraduate project and did not receive external funding), and therefore IRB review was not required. At no point during the study was personally identifiable information collected, and surveys were kept strictly anonymous and confidential.

## Results

As shown in Table 1, of the 41 eligible and interviewed participants, 37 (90.2%) were male, and the median age of participants was 37 years old (25th; 75th percentiles = 30; 43). A total of 33 participants (80.5%) were born in Denmark, while the remaining 8 (19.5%) were born abroad. When asked about their current housing situation, 11 participants (26.8%) reported being homeless, 12 (29.3%) had temporary housing, and 18 (43.9%) resided in a permanent residence. The median age of first injection was 18 years old (17; 22). Seventeen participants (41.5%) had been arrested or charged with a crime in the past six months.

**Table 1:** **Characteristics and drug use behaviors reported by a sample of people who inject drugs at a supervised injecting facility in Copenhagen, Denmark (** ***n*** **= 41)**

Characteristic	n (%) ^b^
**Age (median, 25th; 75th)**	37 (30; 43)
22 – 30	11 (26.8)
31 – 40	14 (34.1)
41 – 49	13 (31.7)
50 – 57	3 (7.3)
**Sex**	
Female	4 (9.8)
Male	37 (90.2)
**Born in Denmark**	
Yes	33 (80.5)
No	8 (19.5)
**Current housing**	
Homeless	11 (26.8)
Temporary	12 (29.3)
Permanent	18 (43.9)
**Age of first injection (median, 25th; 75th)**	18 (17; 22)
12 – 17	16 (39.0)
18 – 23	17 (41.5)
24 – 29	4 (9.8)
30 – 43	4 (9.8)
**Drugs currently used at SIF**	
Cocaine	30 (73.2)
Heroin	25 (61.0)
Methadone	11 (26.8)
Speedball	4 (9.8)
Ritalin	4 (9.8)
Other	8 (19.5)
**Enrolled in treatment** ^**a**^	
Any substance abuse treatment	24 (58.5)
Opioid replacement therapy	20 (48.8)
24 hour treatment	3 (7.3)
Other	6 (14.6)
**Frequency of use at SIF** ^**a**^	
Every day	12 (29.3)
Every couple of days	10 (24.4)
Once a week	3 (7.3)
Every couple of weeks	5 (12.2)
Once a month	3 (7.3)
Less than once a month	5 (12.2)
**Arrested/charged with crime** ^**a**^	
Yes	17 (41.5)
No	22 (53.7)
**Primary site of fixing before SIF**	
Outdoors (street, park, lot, etc.)	25 (61.0)
Own place	23 (56.1)
Other’s place	16 (39.0)
Public washroom	16 (39.0)
Other	2 (4.9)

**Note**: n’s do not sum to 41 and proportions do not sum to 100% due to missing values or the possibility of endorsement of more than one option for some questions.

**Note**: all data collected between February and August, 2013.

^a^= refers to activities or behaviors in the last 6 months.

^b^= n (%) unless otherwise specified.

Also shown in Table 1, cocaine was the most frequently used drug at the SIF, reported by 30 participants (73.2%), followed by heroin, reported by 25 participants (61.0%). Twenty-four participants (58.5%) reported any enrollment in substance abuse treatment in the last six months. The primary injecting location before the SIF opened was outdoors (e.g. street, park, parking lot), reported by 25 participants (61.0%), followed by their own dwelling (56.1%). Since the SIF opened, the majority of participants (61.0%) reported using the facility at least once a week.

As shown in Table 2, the number of people who reported disposing of their used syringes by returning them to the needle exchange or SIF increased from 14 participants (34.1%) before the SIF opened to 36 participants (87.8%) after the SIF opened. The only unsafe disposal method that was still reported by participants after the opening of the SIF was throwing syringes in the garbage, but this behavior decreased from 23 participants (56.1%) before to 5 participants (12.2%) after the opening of the SIF. All other unsafe methods (i.e. dropped them on the ground, gave them to another user, flushed them down the toilet, or other) were reported infrequently before the SIF opened, but were not reported by any participant after the SIF opened. In total, twenty-four individuals (58.5%) reported changing their syringe disposal practices following the opening of the SIF; of those, twenty-three (95.8%) reported changing from not always disposing safely to always disposing safely, (McNemar’s test, *p* < 0.001) while only one individual (4.2%) reported the reverse behavior.

**Table 2:** **Primary locations for disposal of used syringes among a sample of people who inject drugs before and after the opening of a supervised injecting facility in Copenhagen, Denmark (** ***n*** **= 41)**

Disposal mechanism/site	Before SIF opened n (%)	After SIF opened n (%)
Returned to the needle exchange (or SIF)	14 (34.1)	36 (87.8)
Put them in an outdoors sharps container	19 (46.3)	8 (19.5)
Put them in their own sharps container	11 (26.8)	6 (14.6)
Threw them in the garbage	23 (56.1)	5 (12.2)
Dropped them on the ground	5 (12.2)	0 (0.0)
Gave them to another user	2 (4.9)	0 (0.0)
Flushed them down the toilet	4 (9.8)	0 (0.0)
Other	3 (7.3)	0 (0.0)

**Note**: n’s do not sum to 41 and proportions do not sum to 100% because participants may have identified more than one location as primary site of used syringe disposal.

**Note**: all data collected between February and August, 2013.

As shown in Table 3, 31 individuals (75.6%) believed their behaviors had changed since utilizing the services at the SIF. As compared with their behaviors before the SIF opened, 26 participants (63.4%) reported less rushed/stressful injections, 23 participants (56.1%) reported less injecting outdoors, 22 participants (53.7%) reported no longer sharing needles, and 18 participants (43.9%) reported cleaning the injecting site on their skin more often. Other types of behavior change reported by participants are shown in Table 3. The majority (65.9%) did not feel that their frequency of injecting had changed; however, five participants (12.2%) reported a decrease in injecting frequency, while only two participants (4.9%) reported an increase in injecting frequency.

**Table 3:** **Perceived behavior and frequency change among a sample of people who inject drugs at a supervised injecting facility since its opening in Copenhagen, Denmark (** ***n*** **= 41)**

Characteristic	n (%)
**Any perceived behavior change**	31 (75.6)
Less rushed/stressful	26 (63.4)
Less injecting outdoors	23 (56.1)
No longer share needles	22 (53.7)
Clean injection site more often	18 (43.9)
Easier to get vein first time	16 (39.0)
Reuse own needles less often	11 (26.8)
Use clean water more often	11 (26.8)
No longer need help injecting	6 (14.6)
Other	3 (7.3)
**Perceived frequency change**	
No change	27 (65.9)
Decreased (inject less often)	5 (12.2)
Increased (inject more often)	2 (4.9)
Unsure	4 (9.8)

**Note**: n’s do not sum to 41 and proportions do not sum to 100% due to missing values or where participants endorsed more than one option.

**Note**: all data collected between February and August, 2013.

## Discussion

Our results from interviews with a small sample of PWID suggest that use of the SIF in Copenhagen was associated with positive self-reported behavior change and safer syringe disposal practices. Given that the majority of participants reported unstable housing and outdoor injecting before the SIF opened, we conclude that the SIF has been successful in engaging a hard-to-reach population that would otherwise inject primarily outdoors in the Vesterbro area. Furthermore, the majority of participants (61.0%) reported using the SIF at least once a week, which demonstrates that the SIF can serve as a low-threshold health service to engage PWID [27]. Finally, our results suggest that the SIF fills an important gap of other harm reduction interventions in Copenhagen, which do not engage PWID beyond provision of injecting equipment [2].

Three-quarters of our sample believed their behaviors had changed since using the SIF, such as injecting in a less rushed manner, injecting outdoors less often, no longer syringe sharing, and cleaning injecting site(s) more often. Of particular interest is the reduction in public injecting, known to be associated with an elevated risk of blood-borne virus acquisition [28] and overdose [29, 30]. Injecting in public (and in other settings in which maintaining a hygienic drug-using environment is difficult), has also been associated with vascular harm and bacterial infection [31–33]. By providing a nonjudgmental, low-threshold venue, (i.e. use of SIF services requires little bureaucracy, no payment, and is not linked to an obligation of the client to be or to become drug-free) [34] for PWID to consume pre-obtained substances, the Copenhagen SIF has the potential to significantly lower the frequency of deleterious injecting episodes for local drug users. Overall, our finding that users of the SIF have reported a reduction in public injecting, as well as a reduction in publicly discarded syringes, supports the harm reduction framework and goals that the SIF aimed to achieve [30, 31].

Our study adds to the SIF literature, which consists of many reports showing that public drug use has declined since the implementation of SIFs in Vancouver, Sydney, and multiple western European cities [11, 13, 15, 17, 18, 35–39], as well as reports of fewer discarded syringes found in all Swiss cities that have implemented SIFs [2, 14, 40].

Finally, we found associations between use of the SIF and decreased equipment sharing, as well as improved injecting hygiene and technique, in accordance with the existing literature [31, 35, 41–43]. There was no evidence that use of the SIF significantly changed self-reported injecting frequency. This supports previous research demonstrating that SIFs do not result in increased injecting frequency among PWID accessing such facilities [44, 45]. We conclude that, overall, utilization of the SIF has resulted in positive behavior change toward healthier injecting hygiene and reduced risk of bloodborne disease transmission among study participants.

Regarding the capacity of the facility, findings from the Men’s Home’s survey found that approximately 22% of reported injections take place in the users' own homes, and 22% still in the public domain [27]. According to our data, before the SIF opened, 61% of injections took place in the public domain (outdoors) and 56.1% in users’ own homes. Although we did not ask specifically about primary locations of injecting after the opening of the SIF, 56.1% of our sample reported injecting outdoors less frequently. The capacity of the current SIF to accommodate just over half of users’ daily injections is an important finding [27]. Aside from the sheer numbers of injections that the SIF can accommodate, the interviews from the Men’s Home indicate that users choose the SIF for their drug consumption rather than elsewhere because of the security in knowing that there is a health professional to help them should they require medical attention for an overdose or other health issue. Since the data collection phase of both studies, an additional drug consumption room, “Skyen”, or “The Cloud”, has opened nearby in The Men’s Home itself, which can seat eight individuals for injecting and six for smoking, augmenting the overall capacity of SIFs in Copenhagen [27, 46].

To assess whether we reached a sample of PWID similar to that of the Men’s Home’s study, we compared sociodemographic characteristics across the two studies [27]. Their questionnaire, administered in spring 2013, was answered by 56 newly registered users. Their sample comprised 30% female users, while our sample comprises 10% females. Thus, it appears that women may be under-represented in our study. However, the age distribution of the two samples are similar: the participants of the Men’s Home survey were between 22 and 53 years, with a mean age of 38 years, while the ages in our sample spanned 22 years to 57 years, with the mean age at 37 years. We also note that the Men’s Home’s sample comprised a very frequent group of users; nearly half of survey participants presented at the SIF several times a day. Our survey also captured frequent users, as over half came every day or a few times a week, although we also captured infrequent users, as 20% reported coming once a month or less.

One of the primary limitations of our study is a small sample size and limited power to conduct bivariable statistical tests. Further research with a larger sample size is recommended to confirm the observed findings. Moreover, convenience sampling approaches, which were used in this study to recruit participants who presented at the SIF, may prevent generalization to a wider drug-using population in Denmark. As the registry of users at the SIF is completely anonymous, there was no available sampling frame from which to draw for this study. Now that Denmark has implemented additional SIFs in Copenhagen and other cities, it would be beneficial for public health professionals to conduct a multisite study to examine how PWID’s demographics vary among the sites, or how particular programmatic elements of each site have proven successful or unsuccessful.

Our study is subject to a number of additional limitations. First, we relied on self-reported behavior change assessed at one point in time, rather than measuring these variables prospectively at multiple time points, which could introduce bias due to measurement error. The cross-sectional structured behavioral survey also does not allow for inferences about temporal associations and causal pathways between measured factors. However, as our questions were posed in a way that asked about behaviors before and after the opening of the SIF, we believe that our conclusions regarding the association between use of the SIF and behavior change have merit. Second, this study only assessed the short-term effect of the SIF; thus, additional studies should aim to assess longer-term impact on the health behaviors of PWID. Third, a further limitation of our study relates to the sociodemographic characteristics of the sample (e.g., comprised predominately of men). Thus, our results are not entirely representative and may not capture the true lived experiences and behavior changes of women who use the SIF.

Fourth, the survey instrument was not delivered uniformly to all participants; the survey was either self-administered or read aloud by a research assistant, and delivered in either English or Danish. Some members of our research team were not bilingual in English and Danish, and participants may have opted to have the survey read aloud in English even though their primary language was Danish, which could have led to misinterpretations and misreporting of personal information and behavior change. It is therefore possible that some behaviors could be under-reported, over-reported, or misreported. Finally, ascertainment of stigmatized behaviors might have introduced social desirability biases, especially when research assistants read the survey items aloud. This may have been mitigated by providing participants with the opportunity to self-administer the survey, an option they would have most likely chosen had they felt the questions were too sensitive or stigmatizing [47].

## Conclusions

Our findings indicate that utilization of services provided by the first standalone SIF in Copenhagen is associated with self-reported adoption of safer behaviors that reduce harm and promote health among people who inject drugs (e.g., less rushed/stressful injections, less injecting outdoors, and no longer sharing needles). Use of the SIF was also associated with changes in practices that benefit the Vesterbro neighborhood (i.e., safer syringe disposal). As a public health intervention, the SIF in Copenhagen has successfully reached PWID engaging in high-risk behavior, has not led to an increase in overall frequency of injecting, and has resulted in benefits for the greater community. Since the data collection phase of this study, there have been additional SIFs implemented in Copenhagen and across Denmark, which merit their own research and evaluation. However, this pilot peer-reviewed evaluation provides the first evidence that the expansion of Danish SIFs is likely a positive, effective strategy towards improving the health and social equity for people who inject drugs.

## Abbreviations

DCR: Drug consumption room
PWID: People who inject drugs
SIF: Supervised injecting facility.

## References

[1] Haemmig R, Van Beek I, Pates R, McBride A, Arnold K (2005). Supervised Injecting Rooms. Injecting Illicit Drugs.

[2] Broadhead RS, Kerr TH, Grund J-PC, Altice FL (2002). Safer injection facilities in North America: their place in public policy and health initiatives. J Drug Issues.

[3] Hedrich D (2004). European Report on Drug Consumption Rooms.

[4] Dolan K, Kimber J, Fry C, McDonald D, Fitzgerald J, Trautmann F (2000). Drug consumption facilities in Europe and the establishment of supervised injecting centres in Australia. Drug Alcohol Rev.

[5] Schäffer D, Stöver H (2011). Drug Consumption Rooms in Germany: A Situational Assessment by the AK Konsumraum.

[6] Kimber J, Dolan K, van Beek I, Hedrich D, Zurhold H (2003). Drug consumption facilities: an update since 2000. Drug Alcohol Rev.

[7] O’Brien CP (1997). A range of research-based pharmacotherapies for addiction. Science.

[8] O’Brien CP, McLellan AT (1996). Myths about the treatment of addiction. Lancet.

[9] Leshner AI (1999). Science is revolutionizing our view of addiction–and what to do about it. Am J Psychiatry.

[10] Leshner AI (1999). Science-based views of drug addiction and its treatment. JAMA.

[11] Sydney Medically Supervised Injecting Centre (MSIC) (2013). Sydney Medically Supervised Injecting Centre: Fact Sheet.

[12] Drugs and Crime Prevention Committee, Parliament of Victoria (1996). Safe Injecting Facilities: Their Justification and Viability in the Victorian Setting.

[13] Wood E, Tyndall MW, Montaner JS, Kerr T (2006). Summary of findings from the evaluation of a pilot medically supervised safer injecting facility. CMAJ.

[14] Schatz E, Nougier M (2012). International Drug Policy Consortium. Drug Consumption Rooms: Evidence and Practice.

[15] KPMG (2010). Further Evaluation of the Medically Supervised Injecting Centre During Its Extended Trial Period (2007–2011).

[16] Marshall BDL, Milloy M-J, Wood E, Montaner JSG, Kerr T (2011). Reduction in overdose mortality after the opening of North America’s first medically supervised safer injecting facility: a retrospective population-based study. Lancet.

[17] Wood E, Kerr T, Small W, Li K, Marsh DC, Montaner JSG, Tyndall MW (2004). Changes in public order after the opening of a medically supervised safer injecting facility for illicit injection drug users. CMAJ.

[18] DeBeck K, Wood E, Zhang R, Tyndall M, Montaner J, Kerr T (2008). Police and public health partnerships: evidence from the evaluation of Vancouver’s supervised injection facility. Subst Abuse Treat Prev Policy.

[19] Hedrich D, Kerr T, Dubois Arber F, Rhodes T, Hedrich D (2010). Drug consumption facilities in Europe and beyond. Harm Reduction: Evidence, Impacts, and Challenges.

[20] **Drug consumption rooms provide person-based approach to drug use** [http://www.talkingdrugs.org/drug-consumption-rooms-provide-person-based-approach-to-drug-use]

[21] Møller S, Nyhedsbureau B (2012). Fixerum klar til Københavns narkomaner.

[22] Munksgaard PG, Nyhedsbureau B (2012). Let adgang til fixerum glæder kommune.

[23] Sundhedsstyrelsen (2012). National Report (2011 data) to the EMCDDA by the Reitox National Focal Point. Denmark: New Development, Trends, and in-Depth Information on Selected Issues.

[24] **Danish Parliament paves the way towards increased safety and dignity for people who use drugs** [http://www.talkingdrugs.org/idpc/danish-parliament-paves-the-way-towards-increased-safety-and-dignity-for-people-who-use-drugs]

[25] Kristensen FB (2012). Misbrugere strømmer til Vesterbros fixerum.

[26] Stothard B (2012). The Respect Agenda.

[27] Hagensen P (2013). Ifølge Brugerne selv…Den Første Brugertilfredshedsundersøgelse I Forhold Til Københavns Stofindtagelsesrum.

[28] Weeks MR, Clair S, Singer M, Radda K, Schensul JJ, Wilson DS, Martinez M, Scott G, Knight G (2001). High risk drug use sites, meaning and practice: implications for AIDS prevention. J Drug Issues.

[29] Klee H, Morris J (1995). Factors that characterize street injectors. Addiction.

[30] Small W, Rhodes T, Wood E, Kerr T (2007). Public injection settings in Vancouver: physical environment, social context and risk. Int J Drug Policy.

[31] Rhodes T, Kimber J, Small W, Fitzgerald J, Kerr T, Hickman M, Holloway G (2006). Public injecting and the need for “safer environment interventions” in the reduction of drug-related harm. Addiction.

[32] Vlahov D, Sullivan M, Astemborski J, Nelson KE (1992). Bacterial infections and skin cleaning prior to injection among intravenous drug users. Public Health Rep.

[33] Murphy EL, DeVita D, Liu H, Vittinghoff E, Leung P, Ciccarone DH, Edlin BR (2001). Risk factors for skin and soft-tissue abscesses among injection drug users: a case–control study. Clin Infect Dis.

[34] Hedrich D (2005). Data-Collection at Low-Threshold Services for Drug Users: Tools, Quality and Coverage.

[35] Zurhold H, Degkwitz P, Verthein U, Haasen C (2003). Drug consumption rooms in Hamburg, Germany: evaluation of the effects on harm reduction and the reduction of public nuisance. J Drug Issues.

[36] Thein HH, Kimber J, Maher L, MacDonald M, Kaldor J (2005). Public opinion towards supervised injecting centres and the community impact of Sydney medically supervised injecting centre. Int J Drug Policy.

[37] Nickolai M (1997). Evolution of Frankfurt’s approach to the drug problem. Euromethwork.

[38] Kemmesies UE (1999). The Open Drug Scene and the Safe Injection Room Offers in Frankfurt Am Main 1995: Final Report.

[39] Ronco C, Spuhler G, Coda P, Schopfer R (1996). Evaluation for alley-rooms I, II, and III in Basel. Soc Prev Med.

[40] Haemmig RB (1996). Swiss Experiences With Heroin Dispension, Fixer Rooms and Harm Reduction in Prison. Paper Presented at Conference Overlast en Verlichting. Utrecht, the Netherlands.

[41] Wood E, Tyndall MW, Li K, Lloyd-Smith E, Small W, Montaner JSG, Kerr T (2005). Do supervised injecting facilities attract higher-risk injection drug users?. Am J Prev Med.

[42] Kimber J, MacDonald M, van Beek I, Kaldor J, Weatherburn D, Lapsley H, Mattick RP (2003). The Sydney medically supervised injecting centre: client characteristics and predictors of frequent attendance during the first 12 months of operation. J Drug Issues.

[43] Minder MN, Burki CM (1996). Monitoring HIV Risk Behaviours in a Street Agency with Injection Room in Switzerland.

[44] Drucker E (2006). Insite: Canada’s landmark safe injecting program at risk. Harm Reduct J.

[45] Kerr T, Stoltz J-A, Tyndall M, Li K, Zhang R, Montaner J, Wood E (2006). Impact of a medically supervised safer injection facility on community drug use patterns: a before and after study. BMJ.

[46] Hagensen P, Christensen I, Christiansen RK (2013). Stofindtagelsesrummets Første Tre Måneder.

[47] Aquilino WS (1994). Interview mode effects in surveys of drug and alcohol use: a field experiment. Public Opin Q.

